# Application via mechanical dropper alleviates sufentanil-induced cough: a prospective, randomized, single-blinded trial

**DOI:** 10.1186/s13063-019-3274-y

**Published:** 2019-03-15

**Authors:** Minqiang Liu, Zhichao Li, Song Wang, Yong Liu, Xiangpeng Zhong, Renliang He, Fengxian Li

**Affiliations:** 1 Department of Anesthesiology, Shenzhen Third People’s Hospital, No. 29 Bulan Road, Longgang district, Shenzhen, 518112 Guangdong China; 2 Department of Anesthesiology, Zhujiang Hospital of Southern Medical University, No. 253 Middle Industrial Avenue, Haizhu district, Guangzhou, 518112 Guangdong China

**Keywords:** Sufentanil, Cough, General anesthesia, Mechanical dropper

## Abstract

**Background:**

It was reported that prolonging the injection time or diluting administration can reduce the incidence of opioid-induced cough. However, the incidence of sufentanil-induced cough (SIC) via a standardized infusion rate is unclear. A mechanical dropper is an infusion filtering device commonly used for intravenous degassing; it can also be used to administer special drugs due to its temporary storage and dilution effect. This study assesses the effectiveness of administration via mechanical dropper on SIC.

**Methods:**

Two hundred patients undergoing general anesthesia were enrolled. Patients received sufentanil at a strength of 0.3 μg·kg^− 1^ either via T-connector (group C) or by mechanical dropper (group M) at 1 ml·s^− 1^. Cough severity was graded as none (0), mild (1–2), moderate (3–5), or severe (> 5), and the incidence of SIC was evaluated for 5 min after the start of sufentanil injection. Other adverse reactions such as hypotension, hypertension, bradycardia, tachycardia, hypoxemia, vomiting, and aspiration during the induction period of general anesthesia were also observed. The primary outcome was the incidence of SIC. The secondary outcomes were the severity of SIC and other adverse reactions.

**Results:**

The incidence of SIC in group M was significantly lower than that in group C (2% versus 21%, *P* = 0.000), and the prevalence of moderately severe coughing was also statistically different (none in group M versus 11% in group C, *P =* 0.001). However, there were no statistical differences in the incidence of other adverse reactions between two groups (*P* > 0.05).

**Conclusion:**

Sufentanil application via mechanical dropper can significantly alleviate the occurrence of SIC during the induction phase of total intravenous general anesthesia. This method is simple, safe, and reliable, and has wide prospective application for clinical use.

**Trial registration:**

Chinese Clinical Trial Register, ChiCTR-IOR-17011561. Registered on 3 June 2017.

## Background

Opioid-induced cough (OIC) is a common phenomenon during the induction of general anesthesia, and is normally considered to be temporary and not serious [1]. However, sudden, irritating cough may lead to hypertension, tachycardia, anoxia, pneumothorax, and other, even life-threatening, conditions [2, 3], especially for patients suffering from cardiopulmonary dysfunction [4, 5]. Sufentanil is a potent opioid commonly used in clinical anesthesia for its strong analgesic property [6, 7]. The incidence of sufentanil-induced cough (SIC) during the induction of anesthesia has been reported by different studies as being in a range from 16 to 47% [8–10]. Although SIC occurrence can be prevented using pretreatment with drugs such as lidocaine [11], dexmedetomidine [12], or dezocine [13], the existence of notable drug-related side effects has limited their use [14, 15]. Therefore, developing a simple, effective, and nonpharmacological method to counter SIC would be of considerable clinical significance.

A mechanical dropper, also known as a Murphy drip, an infusion set drip cup, or a drip chamber, is an infusion filtering apparatus with a variety of applications. In daily medical care, it can be used for intravenous degassing, observation of the infusion rate, or administration of special drugs when complications are associated with other methods of injection since there is a long intravenous fluid line from this apparatus to the end of the infusion set [16, 17]. In this prospective and randomized study, we compared the effect of sufentanil application via T-connector or mechanical dropper on SIC, hypothesizing that the latter approach can help reduce the incidence of SIC.

## Methods

### Study design and setting

The study was approved by the Medical Ethics Committee of the Shenzhen Third People’s Hospital, Shenzhen, Guangdong, China, on 10 April 2017 (approval number: 2017–059), and was registered in the Chinese Clinical Trial Register (http://www.chictr.org.cn/index.aspx; Registration Number: ChiCTR-IOR-17011561). Written informed consent regarding the study protocol was obtained from all eligible patients. Patients undergoing scheduled general anesthesia who were both aged 18–65 years and classified by the American Society of Anesthesiologists (ASA) physical status classification scheme as belonging to classes I or II were enrolled. The exclusion criteria were those older than 65 years or younger than 18 years, pregnancy, body mass index (BMI) > 30 kg/m^2^, history of bronchial asthma, chronic obstructive pulmonary disease, smoking, upper airway infection in the last 2 weeks, a history of circulatory system diseases or disorders, treatment with angiotensin-converting enzyme inhibitors, impaired kidney or liver function, known hypersensitivity to general anesthetics or opioids, drug abuse, and those who were anticipated to have difficult airway intubation.

### Sample size calculation

**Fig. 1:**
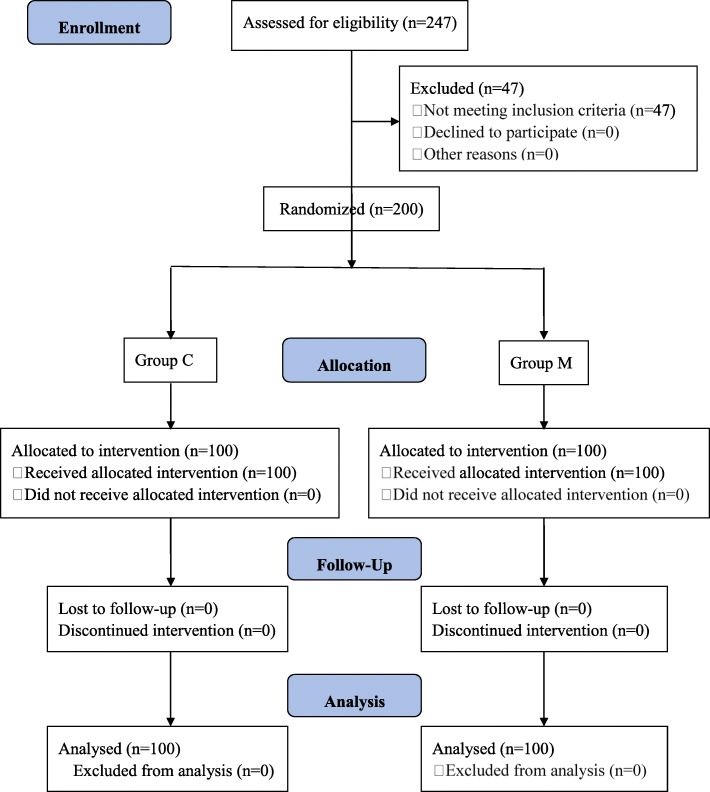
Flow diagram illustrating the structure of the study

The estimation of sample size was based on a 16% reported incidence of SIC following an induction dose of sufentanil of 0.3 μg·kg^− 1^ [9]. A previous study reported a reduction of fentanyl-induced cough via mechanical dropper of approximately 84% [17], so we assumed that this simple method would cause at least an 80% decrease in the incidence of SIC. At *α* = 0.05 and *β* = 0.10, we needed to enroll 93 patients in each group; therefore, we recruited 200 patients to account for possible reduction in sample size due to patients dropping out. The structure of the study is illustrated in Fig. 1.

### Randomization and blinding

Using computer-generated tables of random numbers, patients were assigned to two groups, each containing 100 cases. If a patient’s assigned random number was odd, the patient was assigned to the control group (group C); if it was even, the patient was assigned to the mechanical dropper group (group M). During the general anesthesia induction period, the anesthesiologist giving the anesthetics was cognizant of the patients’ group assignment, but the occurrence of SIC and other parameters was observed and recorded by another anesthesiologist who was not aware of which group the patient had been assigned to and did not take part in the implementation of anesthesia.

### Anesthesia and monitoring

**Fig. 2:**
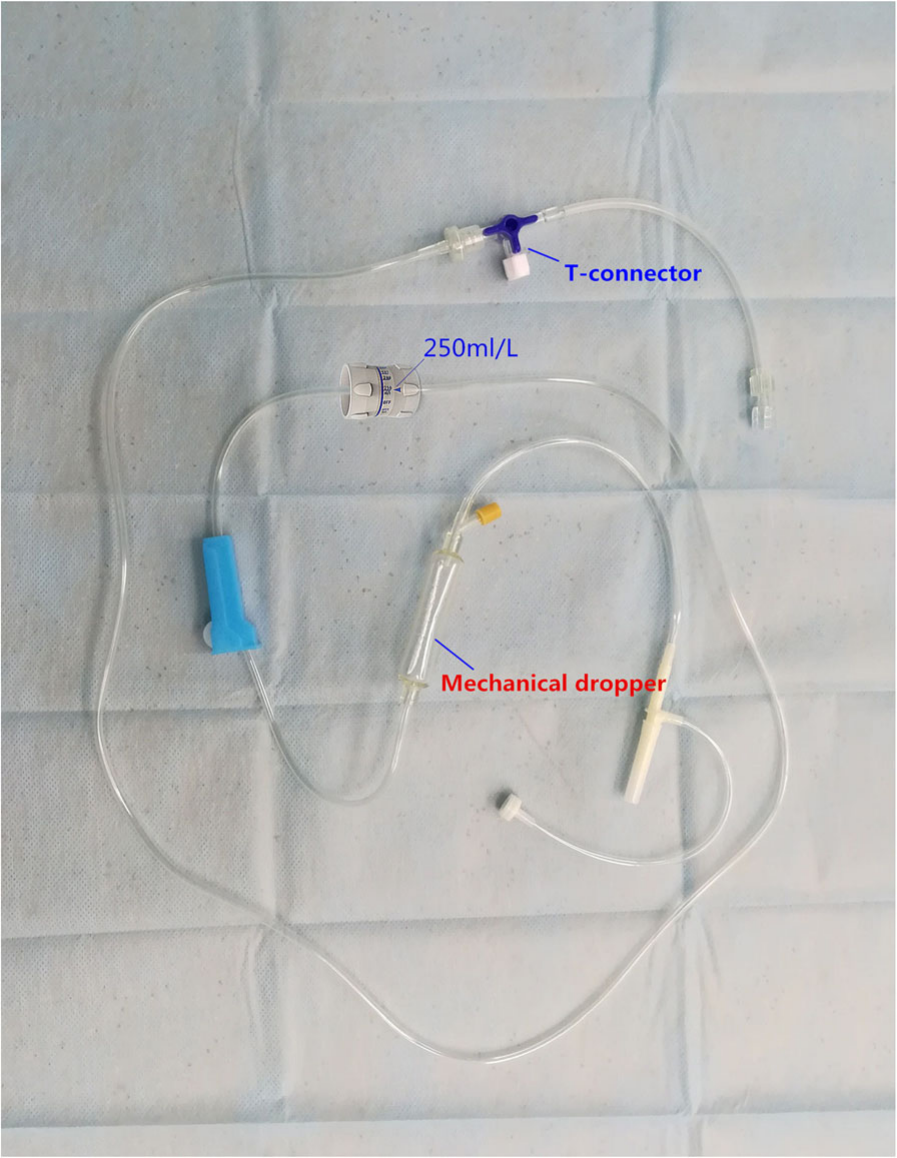
Infusion apparatus: micro-adjustment, mechanical dropper, and T-connector

All patients fasted for 10 h, and no premedication was given before this study. After the patients’ admission, an oxygen gas flow of 2 L·min^− 1^ was given via facial mask. The electrocardiogram (ECG), heart rate (HR), non-invasive blood pressure (NIBP), mean arterial pressure (MAP), pulse oxygen saturation (SPO_2_), end-tidal carbon dioxide partial pressure (P_ET_CO_2_), axillary temperature (T), and bispectral index (BIS) were monitored continuously throughout the procedure. Peripheral venous access was secured using a 20-G venous catheter on the dorsal hand, and normal saline was infused at a rate of 250 ml·h^− 1^ via micro-adjustment of the infusion apparatus (Fig. 2). During the induction phase of general anesthesia, the drip chamber was preloaded with 4 ml of liquid to prevent air from entering the blood vessels, and patients received an injection of sufentanil at a concentration of 0.3 μg·kg^− 1^ (sufentanil citrate, 5 μg·ml^− 1^, diluted with normal saline; Yichang Humanwell Pharmaceutical Co. Ltd, Hubei, China). In group C, sufentanil was injected at a rate of 1 ml·s^− 1^ via T-connector near the venous catheter, while the same volume of normal saline was added to the mechanical dropper at a similar rate. In group M, sufentanil was injected into the dropper and normal saline into the T-connector at 1 ml·s^− 1^. Five minutes after sufentanil administration, the degree of neuromuscular blockade (NMB) was measured, and a sequence of midazolam 0.05 mg·kg^− 1^ (Midazolam Injection; Nhwa Pharmaceutical Co. Ltd, Jiangsu, China), propofol 1.0 mg ~ 1.5 mg·kg^− 1^ (Propofol Medium and Long Chain Fat Emulsion Injection; Fresenius Kabi Austria GmbH, Austria), and cisatracurium 0.15 mg·kg^− 1^ (Cisatracurium Besilate for Injection; Hengrui Medicine Co. Ltd, Jiangsu, China) were injected via T-connector (at 30-s intervals). Endotracheal intubation was then conducted when the bispectral index system baseline remained between 40 and 60, and the first twitch response of a train-of-four stimulation fell to 0 (repeated > 3 times).

### Data collection

Any episode of cough within the 5-min period after the start of sufentanil injection was classified as SIC. The severity of SIC was graded as none (no cough), mild (1–2 instances of cough), moderate (coughing with 3–4 instances), or severe (persistent cough, 5 or more instances) [18]. Vital signs such as the MAP, HR, and SpO_2_ were recorded every 2 min during anesthesia induction (from sufentanil infusion to 5 min after endotracheal intubation). Other adverse reactions such as hypotension (MAP decreased over 20% or NIBP ≤ 90/60 mmHg) [19, 20], hypertension (MAP increased over 20% or NIBP ≥ 140/90 mmHg) [21, 22], bradycardia (HR ≤ 50 beats/min) [23], tachycardia (HR ≥ 100 beats/min) [24], hypoxemia (SpO_2_ < 90%) [25], vomiting (ejecting the contents of the stomach through the mouth) [26], and aspiration (liquid or solid material into the trachea or lung) [27] during the induction period were also recorded.

### Outcomes

The primary outcome was the incidence of SIC. The secondary outcomes were the severity of SIC and other adverse reactions, including hypotension, hypertension, bradycardia, tachycardia, hypoxemia, vomiting, and aspiration.

### Statistical analysis

Statistical analyses were performed using IBM SPSS 13.0 (IBM Inc., Chicago, IL, USA). Data are presented as number or mean ± standard deviation. Group comparisons about age, weight, and height were analyzed using independent *t* test. The frequency of SIC was analyzed by Mann–Whitney *U* test. Between-group comparisons of demographic proportions and the prevalence of adverse reactions were performed using chi-square or Fisher’s exact test. *P <* 0.05 was considered statistically significant.

## Results

A total of 247 patients undergoing general anesthesia were initially assessed for eligibility in this study from July 3, 2017 to December 15, 2017. Forty-seven patients were excluded: 11 patients suffering from upper airway infection in the last 2 weeks, seven patients due to BMI > 30 kg/m^2^, 12 patients due to hypertension, three patients suffering from hypotension, 10 patients due to tachycardia, and four patients for bradycardia. No severe adverse event leading to a termination of the study was observed. Therefore, altogether 200 patients (100 cases in each group) were enrolled in this research (Fig. 1).

### Demographic profile

For sex, age, mean weight, and mean height, the demographic profiles of group C and group M were not statistically different (*P* > 0.05) (Table 1).

**Table 1:** Demographics of the two study groups

Item	Group C	Group M	*P*
Sex (male/female)	45/55	41/59	0.568
Age (years)	38 ± 11	39 ± 10	0.559
Weight (kg)	59 ± 9	58 ± 8	0.457
Height (cm)	163 ± 7	162 ± 7	0.313

Note: Group C, sufentanil injected at a rate of 1 ml·s^− 1^ via T-connector near the venous catheter, while the same volume of normal saline was added to the mechanical dropper at a similar rate; group M, sufentanil injected into the dropper and normal saline into the T-connector at 1 ml·s^− 1^

### Incidence and severity of SIC

The overall incidence of SIC in group M was significantly lower than that in group C (2% of cases in group M versus 21% of cases in group C, *P =* 0.000), and the severity of moderate SIC was also statistically different (no cases in group M versus 11% of cases group C, *P =* 0.001). Furthermore, no cases in group M suffered from severe cough (Table 2).

**Table 2:** Incidence and severity of SIC: primary outcome of the study

SIC incidence (%)	Group C	Group M	*P*
None	79	98^**^	0.000
Mild	8	2	0.052
Moderate	11	0^**^	0.001
Severe	2	0	0.095
Total incidence	21	2^**^	0.000

Note: Group C, sufentanil injected at a rate of 1 ml·s^− 1^ via T-connector near the venous catheter, while the same volume of normal saline was added to the mechanical dropper at a similar rate; group M, sufentanil injected into the dropper and normal saline into the T-connector at 1 ml·s^− 1^. *SIC* sufentanil-induced cough

Compared with group C, ^*^P < 0.05, ^**^P < 0.01

### Other adverse reactions

There were no statistical differences in the prevalence of hypotension, hypertension, bradycardia, or tachycardia between the two groups (*P* > 0.05). No patient suffered from hypoxemia, vomiting, or aspiration during the general anesthesia induction period (Table 3).

**Table 3:** Other adverse reactions: secondary outcomes of the study

Item	Group C	Group M	*P*
Hypotension (%)	19	17	0.713
Hypertension (%)	6	7	0.774
Bradycardia (%)	14	12	0.674
Tachycardia (%)	9	9	1.000
Hypoxemia (%)	0	0	1.000
Vomiting (%)	0	0	1.000
Aspiration (%)	0	0	1.000

Note: Group C, sufentanil injected at a rate of 1 ml·s^− 1^ via T-connector near the venous catheter, while the same volume of normal saline was added to the mechanical dropper at a similar rate; group M, sufentanil injected into the dropper and normal saline into the T-connector at 1 ml·s^− 1^

## Discussion

The mechanisms responsible for SIC are complicated, and include low compliance of the chest wall, inhibition of central sympathetic activity, histamine release, neurogenic inflammation, and tracheal hypersensitivity reaction, among others [28–31]. In previous studies, it was reported that the incidence of SIC was dose related [10, 32], thus reducing the dosage of sufentanil, diluting it, or prolonging the injection time may help to alleviate SIC. However, excessive medication dilution or a long series of manual injection times may reduce the adherence of anesthesiologists, even the controllability of anesthesia. Therefore, finding a simple way to treat SIC without affecting the quality of anesthesia is highly desirable.

Our study evaluates an ordinary method to apply sufentanil via mechanical dropper. Here, we found that our procedure successfully reduced SIC during total intravenous general anesthesia without any other medical treatment required during induction. There are three possible explanations for this inhibitory effect. First, when sufentanil was added to the mechanical dropper, it was diluted by the original liquid present in the dropper, the fluid coming from the infusion bag, and the mixture continuously dropping down to the flow tube [17]. In this research, the average patient weight in both groups was approximately 60 kg, with an induction dose of sufentanil of 0.3 μg·kg^− 1^ and an initial concentration of 5 μg·ml^− 1^, the average dosage was around 18 μg, and the mean volume was 3.6 ml. Since there was 4 ml of liquid in the mechanical dropper and 10 ml within the infusion line below, and continuous fluid coming from the infusion bottle, the mean concentration of sufentanil arriving in the blood vessels was less than 1 μg·ml^− 1^. As a result, the direct stimulation of sufentanil on the circulatory system was reduced. Second, accompanying continuous dilution, the osmolality and pH of sufentanil became close to the normal saline solution before entering the peripheral veins through a long intravenous fluid line. As a result, the stimulation from the vein to irritant receptors in tracheal smooth muscle tissues and C fiber receptors in pulmonary vessels was reduced, and the sudden vocal cord closure triggered by laryngeal muscle spasticity was thereby alleviated [28, 31]. Third, the drug infusion rate was limited via a long fluid line. Considering the differences in vascular conditions between individuals, we used micro-adjustments to standardize the infusion rate at 250 ml·h^− 1^; with an initial liquid volume of 14 ml from the dropper to the venous catheter, it took about 5 min for the sufentanil to completely enter the patient’s body. For this reason, the actual injection time of the sufentanil was prolonged significantly, resulting in a much lower peak plasma concentration. Consequently, dose-related adverse reactions such as cough were alleviated, and a lower incidence of severe SIC was observed. Moreover, we found no differences in the prevalence of circulatory or respiratory complications such as hypotension, hypertension, bradycardia, tachycardia, or hypoxemia between the control and experimental groups, which suggests that sufentanil application via mechanical dropper is not associated with these serious side effects.

There are some limitations to our findings here. First, we set a low infusion rate to avoid individual differences in vascular conditions from affecting the treatment. Consequently, the total sufentanil infusion time was much longer in group M than is typical in clinical practice. Second, since the onset time of intravenous sufentanil was 1–3 min and the time to peak effect was around 5 min [7], we evaluated the incidence of SIC within the 5-min period after the start of sufentanil injection. However, the actual injection time in group M was about 5 min, meaning SIC may still appear several seconds to minutes after the real injection was finished. A longer observation period of SIC, such as 10 min after the start of sufentanil injection, could prevent the possible bias. Third, the interactive effects of other medications often given with sufentanil were not tested. Sedatives such as midazolam or propofol were often administered before the use of opioids to reduce the patient’s discomfort, and our treatments did not include these. Finally, the average age of patients enrolled in our research was around 40 years, but age is believed to be an important confounding factor in OIC [33]. Elders, children, infants, and larger-scale treatment that also assessed the optimal sequence of drug administration would be beneficial to further verify the effectiveness of the application of sufentanil via mechanical dropper.

## Conclusions

This study reports that the application of sufentanil via mechanical dropper can effectively alleviate SIC during total intravenous general anesthesia induction. This method is simple, safe, and reliable, and is suitable for poorly equipped hospitals in developing countries.

## Abbreviations

ASA: American Society of Anesthesiologists
BIS: Bispectral index
ECG: Electrocardiogram
HR: Heart rate
MAP: Mean arterial pressure
NIBP: Non-invasive blood pressure
NMB: Neuromuscular blockade
P_ET_CO_2_: End-tidal carbon dioxide partial pressure
SIC: Sufentanil-induced cough
SPO_2_: Pulse oxygen saturation
T: Temperature
